# Clinical study of a spray containing birch juice for repairing sensitive skin

**DOI:** 10.1007/s00403-023-02588-4

**Published:** 2023-03-24

**Authors:** Xiaohong Shu, Shizhi Zhao, Wei Huo, Ying Tang, Lin Zou, Zhaoxia Li, Li Li, Xi Wang

**Affiliations:** 1 Cosmetics Evaluation Center, West China Hospital, Sichuan University, Chengdu, Sichuan People’s Republic of China; 2 Department of Dermatology, West China Hospital, Sichuan University, No. 37 Guo Xue Xiang, Chengdu, Sichuan 610041 People’s Republic of China; 3 Yoseido (Shanghai) Cosmetics R&D Co., Ltd., Shanghai, People’s Republic of China

**Keywords:** Birch juice, Sprays, Sensitive skin, Clinical efficacy

## Abstract

Sensitive skin is described as an unpleasant sensory response to a stimulus that should not cause a sensation. Sensitive skin affects an increasing proportion of the population. Sixty-seven participants who tested positive to lactic acid sting test were recruited and randomized into two groups to observe the clinical efficacy and safety of a new birch juice spray for repairing sensitive skin. One group used test spray A, while the other group used spray B as a control. Both groups were sprayed six times daily for 28 days. Noninvasive testing instruments were used to measure stratum corneum hydration, sebum content, transepidermal water loss rates, skin blood perfusion and current perception threshold before and after using spray. Facial images were captured by VISIA-CR, and the image analysis program (Image‐Pro Plus) was used to analyze these to obtain the redness value of the facial skin. Moreover, lactic acid sting test scores and participants’ self-assessments were also performed at baseline, week 2 and week 4. Both sprays A and B significantly decreased the lactic acid sting test score, transepidermal water loss rates, skin blood perfusion, and redness, while increasing the stratum corneum hydration. Compared to spray B, spray A increased sensory nerve thresholds at 5 Hz and decreased the transepidermal water loss rates, skin blood perfusion, and lactic acid sting test score. Sprays containing birch juice improved cutaneous biophysical properties in participants with sensitive skin.

## Introduction

Sensitive skin refers to a high reaction state occurring under physiological or pathological conditions, mainly in the face, manifested as the skin being prone to burning, tingling, itching, and tension when stimulated by physical, chemical, physiological, and psychological factors, accompanied or not accompanied by erythema, scales, telangiectasia and other objective signs [1, 2]. Sensitive skin symptoms often occur repeatedly and seriously affect the appearance of a patient, causing great physical and psychological pressure [3].

Factors affecting sensitive skin include external and internal factors. External factors include environmental factors (humidity, temperature/climate change, environmental pollution, ultraviolet radiation, wind) and lifestyle (cosmetics, diet, and alcohol), while internal factors include sex, age, hormone level, emotion, stress, and genetic factors [4, 5]. Recently, the number of people with sensitive skin has increased due to air pollution and the diversified use of cosmetics. Approximately 50% of people claim that they have sensitive skin, and this proportion is gradually rising [6–8]. Therefore, nursing sensitive skin is increasingly important.

The pathophysiology of sensitive skin includes damage to the skin barrier, an enhanced immune response, and heightened neurosensory sensitivity. In the Muizzuddin classification, skin sensitivity can be classified into three types: skin barrier damage type, inflammatory response type, and nerve hyper-response type [4]. These types can influence and interact with each other, suggesting that in nursing sensitive skin, considering the three dimensions is crucial. At present, studies on sensitive skin care have been more extensive [9], although reports on the comprehensive clinical care of sensitive skin from multiple dimensions are few.

Birch juice, a colorless and transparent or can be slightly yellow fresh juice from the birch, contains rich nutrients and biologically active material. It is often regarded as a kind of simple and quick beverage, and it also has medicinal and cosmetic effects [10–14]. To date, birch juice contains 11 kinds of fatty acids, 18 kinds of amino acids, 4 kinds of vitamins and 18 kinds of mineral elements as well as compounds of nicotinic acid, essential oil, betula bud acid, saponin, cell division elements, growth elements, sulfur ammonia elements, and pyridoxine [15–17]. Among them, amino acids, fatty acids, and vitamins play an important role in maintaining the skin barrier function, reducing inflammation, skin moisturizing, wound healing, and whitening [18–22]. The rich mineral elements in birch juice are also very valuable for skin care [23, 24]. However, the clinical application of birch juice for repairing sensitive skin has not been reported. Thus, this study used multidimensional methods to evaluate the repair effect of a moisturizing spray containing natural birch sap on sensitive skin and compared it with commercially available sprays.

## Materials and methods

### Study design

This randomized, double-blind, clinical study was conducted. The research protocol was reviewed and approved by the Institutional Ethics Committee. All participants provided informed consent.

### Study participants

Altogether, 67 people were selected, of which 33 were assigned to the Group A and 34 were in the Group B by random software distribution. The inclusion criteria were as follows: aged between 18 and 60; in good health; with positive lactic acid stimulation test and experiences skin discomfort when the season changes in previous years; participated in the study voluntarily and signed the informed consent form; and able to strictly comply with the requirements of the study protocol, use the product as required, and complete follow-up. The exclusion criteria were as follows: pregnant or lactating women; with skin diseases (such as psoriasis, eczema, psoriasis, and skin cancer), evident erythema, sunburn, wound, wear, and tattoo, in or near the test area; have participated in other clinical studies or been treated by dermatologists within the last 3 months; and used any other anti-allergy products within the last 3 months. Rejection and termination were considered when the participants requested to discontinue the test or when adverse reactions occurred, respectively.

### Test spray

The test product (A) was a spray containing natural birch juice and birch bark extract. The control product (B) was a spray containing thermal spring water on the market. The main ingredients of spray A and spray B are shown in Table 1.

**Table 1:** Main ingredients of spray A and spray B

No	Name	Main functional ingredient
1	Spray A	Betula alba juice ≥ 88% Betula alba juice: Mineral elements (calcium, potassium, magnesium, manganese, sodium, zinc, barium, boron, strontium, ferrum, silicon, cuprum, cobalt, nickel, cadmium), amino acid (lysine, alanine, threonine, cystine, histidine, serine, valine, *Isoleucine*, methionine, leucine, glycine, phenylalanine, arginine, thyroxine, tryptophan, proline, aspartic acid, glutamic acid), aliphatic acid, monosaccharide, Vitamin (Vitamin C, Vitamin B1, Vitamin B2, Vitamin H)
2	Spray B	Silice, trace elements (Al, Ba, Li, Sr, Zn), cations (Co^2+^, Mg^2+^, Na^+^), anions (HCO_3_^−^, SO_4_^2−^, Cl^−^)

### Treatments

All participants were asked to use the spray six times a day 15–20 cm from the face by pressing the pump head in a circular motion and spraying on the face without it. One group received spray A, and the other received spray B. To ensure dose compliance, the volume of residual spray in the container was examined at each follow-up. Assessments of skin biophysical properties were performed at the indicated times.

### Evaluation method

The VISIA-CR 4.1 skin analysis imaging system (Canfield Imaging Systems, Fairfield, NJ, USA) equipped with a Canon EOS-5Ds Mk III SLR camera was used to capture images from the front and left and right sides at 45°. The images were captured under the following lighting conditions: standards 1 and 2, cross-polarized, parallel-polarized, and UV.

Tewameter^®^ TM300 (MPA, Courage-Khazaka Electronic GmbH, Koln, Germany) was used to investigate transepidermal water loss rates (TEWL) and Corneometer^®^ CM825 (MPA, Courage-Khazaka Electronic GmbH, Koln, Germany) was used for detecting stratum corneum (SC) hydration. TEWL was measured in triplicate on the perioral areas for each subject. SC hydration was tested thrice on the cheekbones. These values were calculated based on their average.

Skin blood flow in the cheek was monitored by laser Doppler flowmetry (PeriFlux 5000; Perimed AB, Sweden). The amount of blood perfusion was used to measure skin microcirculation, and the higher the redness of the participant’s facial skin, the greater the value.

The current perception threshold (CPT) test was conducted using a Neurometer^®^ CPT/C quantitative sensory nerve detector (Neurotron Inc., Baltimore, MD, United States) via a standardized automatic double-blind test method. The lower maxillary branch of the trident meridian was tested. The electrode water was placed horizontally in the middle of one side of the mandibular bone. The Neurometer^®^ CPT/C at three different frequencies (2000 Hz, 250 Hz, and 5 Hz) is an electric current generator that provides selective stimulation for three subpopulations of sensory nerve fibers in the skin. Typical skin sensory nerves are composed of three main subgroups of nerve fibers: Aβ fibers, which conduct skin sensation and pressure; Aδ fibers, which conduct temperature, pressure, and acute pain; and C fibers, which conduct temperature and chronic pain. The current perception threshold (CPT) of the skin can be quantitatively measured using a CPT/C neurometer which reflects the skin’s sensitivity to stimulation; the lower the CPT value, the more sensitive the sensory nerves in the skin are to stimuli, and vice versa [25].

The photographs under Visia-CR cross-polarized light were evaluated for redness value assessed using an image analysis program (Image Pro-plus 7.0; Media Cybernetic Inc., Rockville, MD, USA). The software quantified the color of the facial skin using the *L*, *a*, and *b* color spaces, where *L* was lightness, a denoted redness, and *b* indicated yellowness. The higher the *a*-value, the reddish the skin.

The lactic acid sting test (LAST) was conducted as follows: 50 µl of 10% lactic acid solution was applied on one side of the nasolabial sulcus and cheek, and distilled water was applied on the other side, randomly left and right. The participants were asked about regarding their self-conscious symptoms at 2.5 min and 5 min, respectively, and scored on a 4-point scale (0 for no stinging, 1 for slight stinging, 2 for moderate stinging, and 3 for strong stinging). The two scores were then summed, and a total score ≥ 3 was classified as the LAST positive participant.

In the participants’ subjective evaluation, they evaluated their skin condition at the second and fourth week of follow-up. To evaluate whether the skin discomfort caused by changing seasons is less than in previous years, the improvement standard is divided into five levels as follows: more evident discomfort, no change, slightly reduced, reduced, and significantly reduced. The number of participants with significant reduction, reduction and slight reduction is the reduction rate. The evaluation parameters also included prevented the occurrence of new sensitives, repaired sensitivity and no irritation. The score was divided into the following five levels: completely disagree, somewhat disagree, disagree, not disagree, somewhat agree, and completely agree. The total number of participants of complete agreement and some agreement is the agreed rate. In the week 4, the satisfaction evaluation was conducted, and the standard was divided into four levels: very satisfied, satisfied, general, and dissatisfied. The number of very satisfied and satisfied cases is the satisfaction rate.

The parameters were evaluated before application and after 2 and 4 weeks of use. The testing environment was maintained at a constant 22 ℃ ± 1 ℃ and 50% ± 5% humidity. Before sampling, the participants sat in a temperature-controlled room quietly for 20 min.

### Statistical analysis

The data were analyzed using SPSS version 19.0 and are expressed as the mean ± standard deviation. The data that followed approximate positive distribution were compared using mixed linear models. For participants’ self-assessment, the Wilcoxon test was used to compare W0 at different time points in the same group, and the Mann–Whitney *U* test was used to compare the same time difference between groups. Significance was set at *p* < 0.05.

## Results

### Screening results of participants

Overall, 67 participants were included in the study, of which 33 were in the Group A, and 34 were in the Group B. Two participants in the Group A and one participant in the Group B could not complete the entire study due to personal reasons. Finally, 31 participants in the Group A, and 32 participants in the Group B completed the trial. The average age of the participants in the Group A was 37.9 ± 12.5 years, while that of the Group B was 39.7 ± 12.7 years. The baseline data for participants were comparable between the two groups (Table 2).

**Table 2:** The baseline data

Items	Group A (*n* = 31)	Group B (*n* = 33)	*P* value
Age, years	37.94 ± 12.50	39.76 ± 12.72	0.554
Sex, female (%)	31 (100)	33 (100)	1.000
Hydration value	60.62 ± 12.44	60.80 ± 12.84	0.748
TEWL	20.23 ± 3.31	17.57 ± 4.94	0.013
Blood perfusion	89.81 ± 62.62	112.49 ± 89.37	0.308
Redness value	6.56 ± 3.80	7.26 ± 3.71	0.803
Current perception threshold (2000 HZ)	105.18 ± 22.32	105.08 ± 27.25	0.975
Current perception threshold (250 HZ)	29.03 ± 15.24	31.12 ± 13.75	0.475
Current perception threshold (5 HZ)	16.52 ± 11.88	13.38 ± 8.08	0.857
Last	4.45 ± 0.72	4.33 ± 0.60	0.326

### Improvement of skin barrier function

**Fig. 1:**
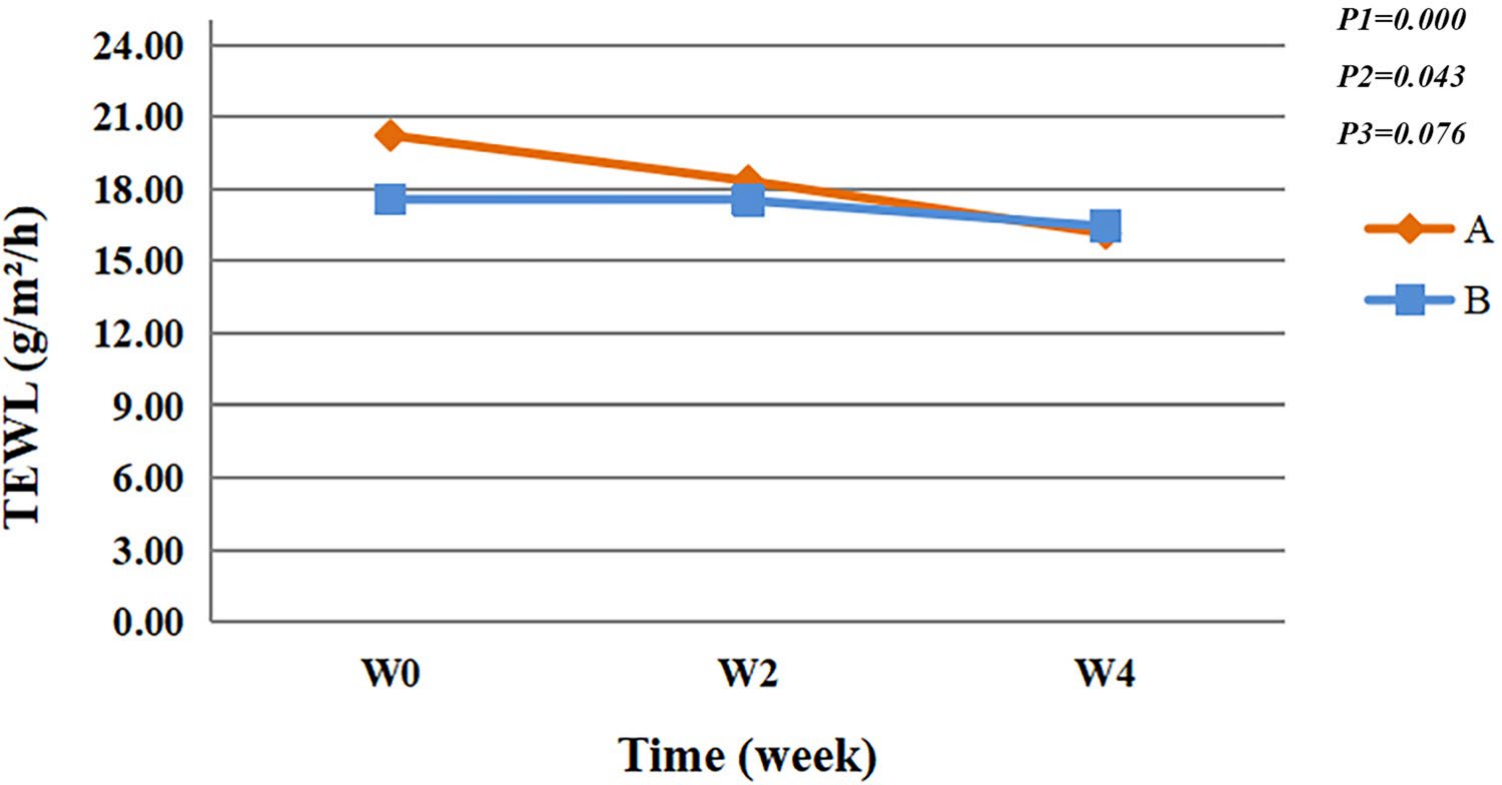
Transepidermal water loss value. P1 = time effect; P2 = group effect; P3 = interaction group × time

Both groups showed significant reductions on the TEWL value over time (*F* = 9.156, *P* < 0.05). After 4 weeks of using spray, the TEWL value was significantly lower than baseline (*P* < 0.05) (*F* = 4.151, *P* < 0.05), and the TEWL value in group A was significantly lower than that in group B (estimate: A:0, B:0.299). No significant interaction effects for Group and times were observed (*F* = 2.625, *P* > 0.05) (Fig. 1).

**Fig. 2:**
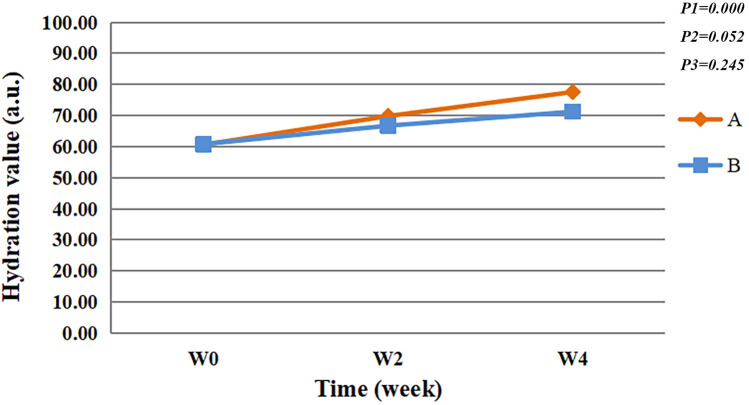
Hydration value. P1 = time effect; P2 = group effect; P3 = interaction group × time

As the intervention time increases, the hydration value of participants in the two groups were significantly improved (*F* = 24.709, *P* < 0.05), significant differences were found at W0 and W2 (*P* < 0.05), W0 and W4 (*P* < 0.05). No significant difference is found between the groups (*F* = 3.827, *P* > 0.05).There was no interaction effect between time points and groups (*F* = 1.423, *P* > 0.05)(Fig. 2).

### Improvement of facial redness

**Fig. 3:**
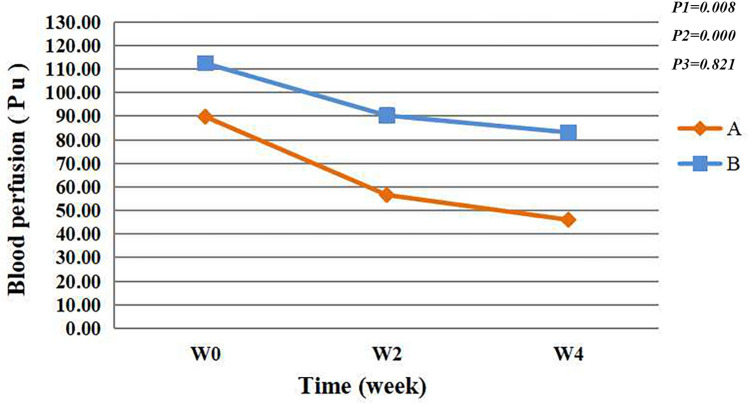
Blood perfusion. P1 = time effect; P2 = group effect; P3 = interaction group × time

A laser Doppler blood flow meter is used to measure the amount of skin microcirculation blood perfusion; the more serious the skin redness is, the higher the value of blood perfusion. The blood perfusion decreased significantly over time (*F* = 5.053, *P* < 0.05), significant differences were found at W0 and W4 (*P* < 0.05). Significant between-group differences were seen (*F* = 12.733, *P* < 0.05), group A had significantly lower blood perfusion compared to the group B. (estimate: A: 0, B: 37.107); there was no interaction effect between time points and groups (*F* = 0.197, *P* > 0.05)(Fig. 3).

**Fig. 4:**
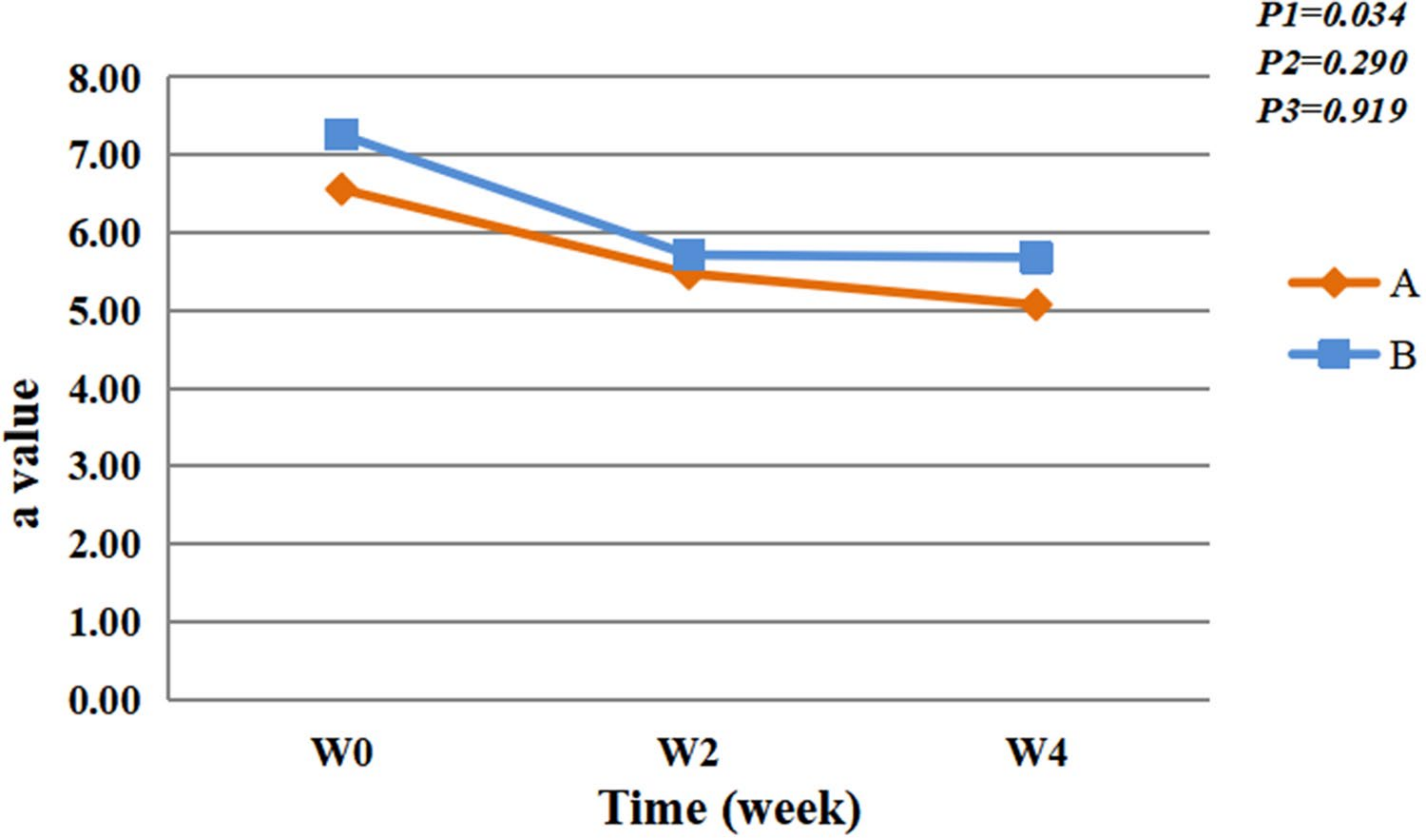
A value. P1 = time effect; P2 = group effect; P3 = interaction group × time

**Fig. 5:**
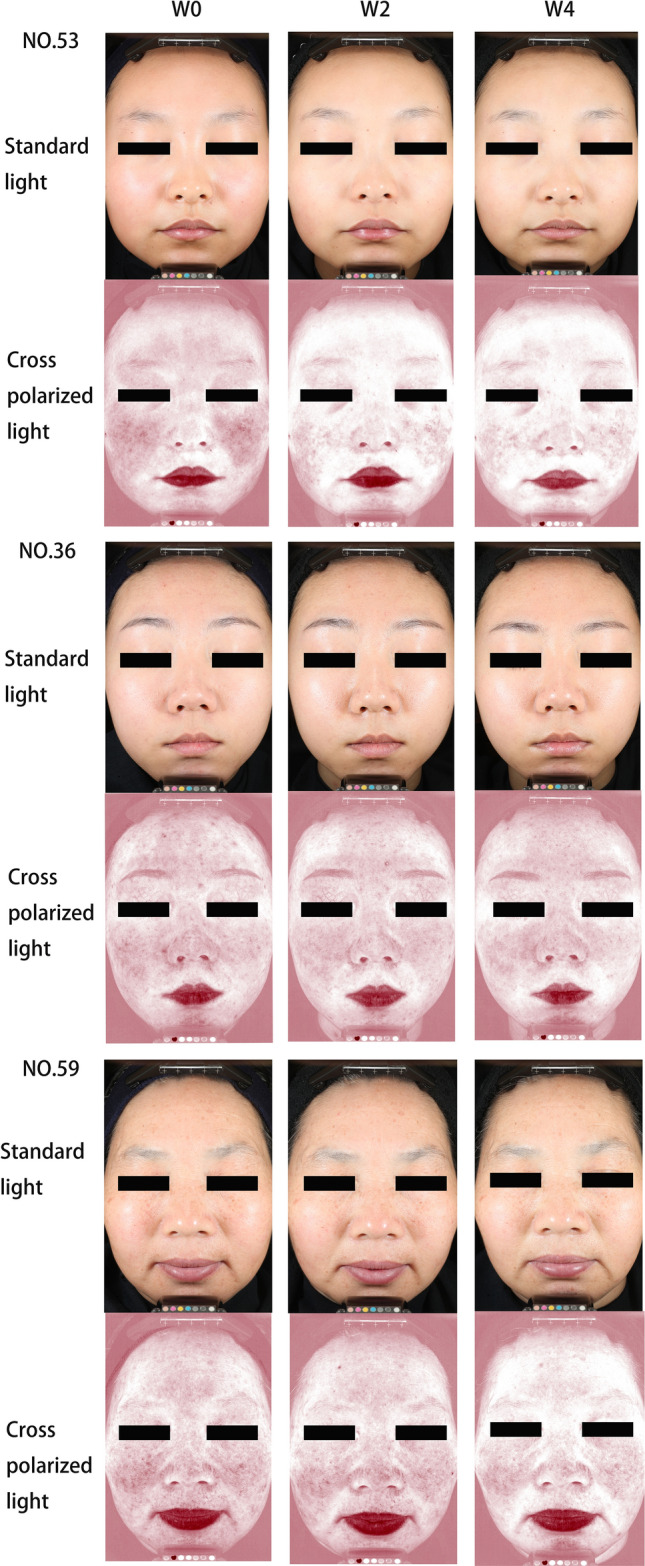
Representative images of three participants before and after the application of spray A

Before and after using the spray, an image analysis program was employed to analyze the photos under the cross-polarized light of Visa CR, and obtain the value of facial redness. The facial redness of the participants in the two groups decreased significantly over time (*F* = 3.485, *P* < 0.05). There was no group significant difference (*F* = 1.124, *P* > 0.05) and group × time interaction effect (*F* = 0.084, *P* > 0.05) (Fig. 4). Figure 5 depicts the comparison of three participants before and after using spray A.

### Improvement of sensory nerve sensitivity

#### Measurement results of sensory nerve stimulation threshold

**Fig. 6:**
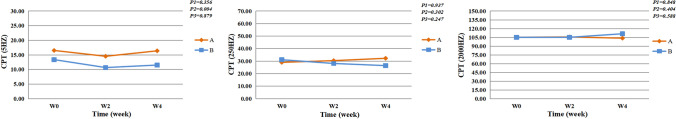
Current perception threshold. P1 = time effect; P2 = group effect; P3 = interaction group × time

At 5 Hz, there was no difference over time (*F* = 1.042, *P* > 0.05) and no effect of interaction between the time and group (*F* = 0.129, *P* > 0.05). Significant differences were observed between the groups for the sensory threshold at 5 Hz (*F* = 8.563, *P* < 0.05), which in group A was higher than that in group B (estimates, A: 0, B: − 4.87). At 250 Hz, there was no significant difference in time effect (*F* = 0.065, *P* > 0.05), group effect (*F* = 1.070, *P* > 0.05), and time × group effect (*F* = 1.414, *P* > 0.05). At 2000 Hz, there was no effect over time (*F* = 0.165, *P* > 0.05), no difference between groups (*F* = 0.404, *P* > 0.05), and no interaction between the two variables (*F* = 0.534, P > 0.05) (Fig. 6).

#### Experimental results of LAST

After 2 weeks and 4 weeks of using sprays A and B, respectively, the LAST score of the participants in the two groups decreased gradually, and a significant difference was noted compared to baseline (*P* < 0.05). The comparison between groups indicated that at week 4, the scores of LAST in group A were more significantly reduced than those in group B (*P* < 0.05) (Table 3).

**Table 3:** The results of the lactic acid sting test at week 4

Test time	Group A	Group B
W0	4.45 ± 0.72	4.33 ± 2.60
W2	3.68 ± 1.22*	3.79 ± 2.06*
W4	2.84 ± 1.16^*,#^	3.42 ± 1.00*

Compared with week 0; *P < 0.05. Compared with Group B; ^#^*P* < 0.05

### Subjective evaluation

#### Subjective evaluation of the participants

Before and after using the spray, the participants self-assessed their skin condition. After using spray A for 4 weeks, 83.87% of the participants believed that their skin discomfort caused by changing seasons was reduced compared with that in previous years. After 4 weeks of using spray B, 78.79% of the participants felt less skin discomfort caused by changing seasons compared with previous years. There was no significant difference between the groups (Table 4).

**Table 4:** Subjective evaluation of skin discomfort due to the change of season at week 4

Group	Compared with previous years, whether the skin discomfort caused by the change of season was reduced						
	More obvious	No change	Slightly reduced	Reduced	Significantly reduced	Reduced ratio (%)	*P*
Group A	0 (0%)	5 (16.13%)	15 (48.39%)	7 (22.58%)	4 (12.9%)	83.87	0.595
Group B	0 (0%)	7 (21.21%)	14 (42.42%)	12 (36.37%)	0 (0%)	78.79	

^a^The number of participants with significant reduction, reduction, and slight reduction is defined as the reduction rate

As shown in Table 5, the participants believed that the spray prevented the occurrence of new sensitivities and repaired sensitive skin at W4 visit, the percentage of A and B for both groups was 96.78% and 87.88%, respectively. The difference between the two groups was significant. Most participants in both groups agreed that spray could repaired sensitive skin (96.77% and 93.94% in Group A and Group B, respectively). Significant differences were found between the two groups. More than 96% of the participants rated the products as non-irritating in both the groups, no significant differences between groups.

**Table 5:** Participants' self-assessment at week 4

	Group	Completely disagree	Somewhat disagree	Not disagree	Somewhat agree	Completely agree	Agreed rate (%)	*P*
To prevent the occurrence of a new sensitivity	Group A	0 (0%)	0 (0%)	1 (3.23%)	8 (25.81%)	22 (70.97%)	96.78	0.001^#^
	Group B	0 (0%)	0 (0%)	4 (12.12%)	20 (60.61%)	9 (27.27%)	87.88	
Repaired sensitive skin	Group A	0 (0%)	0 (0%)	1 (3.23%)	10 (32.26%)	20 (64.52%)	96.77	0.028^#^
	Group B	0 (0%)	0 (0%)	2 (6.06%)	19 (57.58%)	20 (36.36%)	93.94	
No skin irritation	Group A	0 (0%)	0 (0%)	0 (0%)	1 (3.23%)	30 (96.77%)	100	0.982
	Group B	0 (0%)	0 (0%)	1 (3.03%)	0 (0%)	32 (96.97%)	96.97	

^#^*P* < 0.05

^a^The total number of participants expressing complete agreement and some agreement is the agreed rate

Compared with Group B

**Fig. 7:**
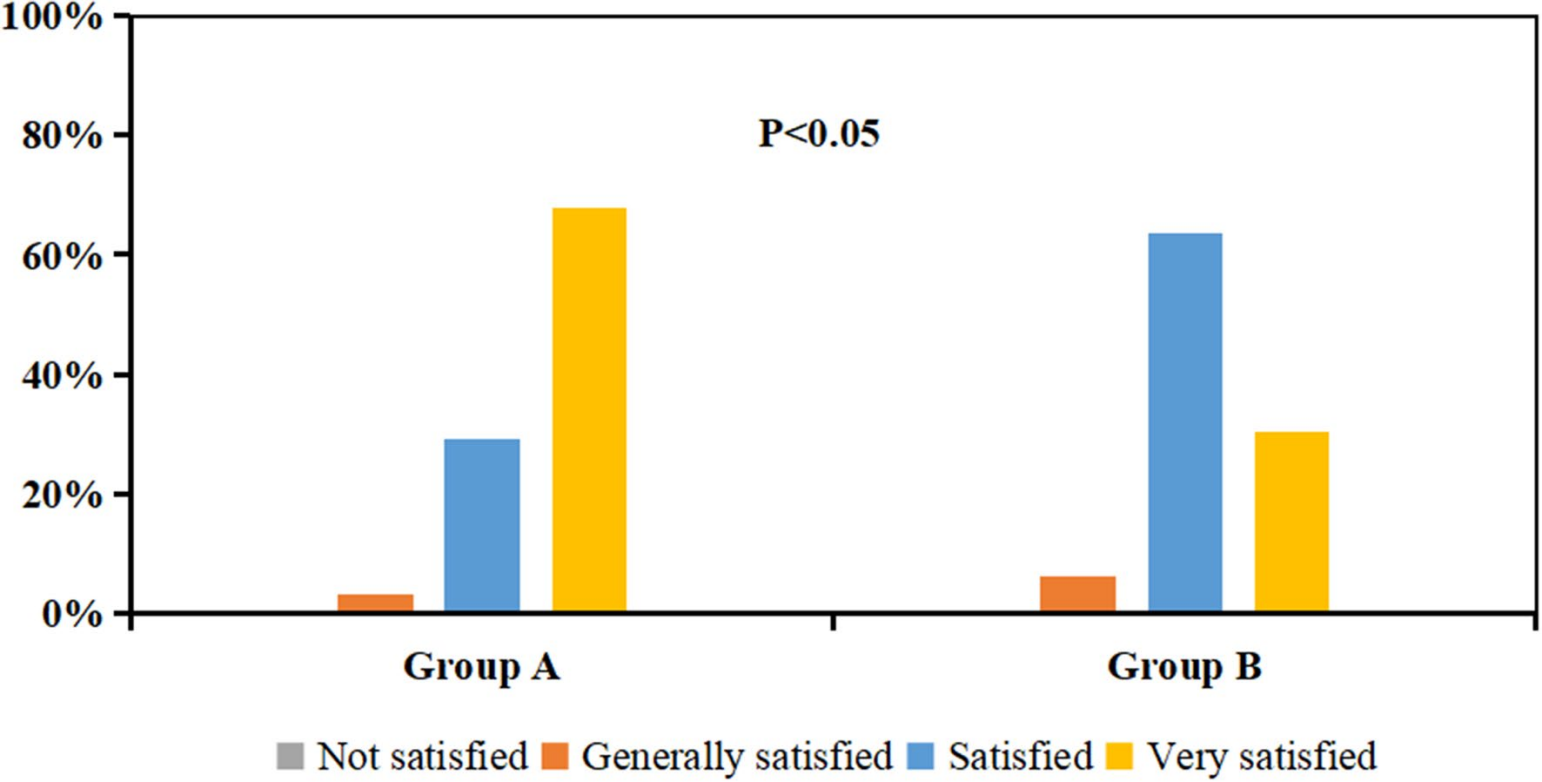
Percentage of participants with different satisfaction scores in groups A and B at week 4. P: Group A compared to Group B

As shown in Fig. 7, the satisfaction rate of the participants in group A reached 96.77% after 4 weeks of using spray A, while that of the participants in group B reached 93.94% after 4 weeks of using spray B. There was a significant difference in satisfaction rate between the two groups.

## Discussion

After the use of the birch spray by the Group A, SC hydration increased significantly, and the TEWL and redness degree were significantly reduced, while the CPT was increased, and the LAST score was significantly decreased. Thus, the spray containing natural birch juice can effectively improve the skin barrier function, relieve discomfort such as redness and tingling caused by inflammation, and reduce the sensory nerve sensitivity of the skin.

The epidermis is the interface where the human body makes contact with the external environment, and one of its main functions is the barrier function. Impaired skin barrier function is a common pathological mechanism of sensitive skin [4]. The barrier function of the epidermis is closely related to the various lipids, proteins, water, inorganic salts, and other metabolites of the epidermis. The cuticle protects the body, is an important penetration barrier of the skin, and can hinder the loss of percutaneous evaporation of water. TEWL reflects the amount of water evaporation from the skin surface and is therefore an important indicator for evaluating skin barrier function [26]. After 4 weeks, the TEWL value was significantly lower (*P* < 0.05) (Fig. 1), and the SC hydration was significantly different compared to the baseline (*P* < 0.05) (Fig. 2). Hence, the use of natural birch juice-containing spray significantly improved the skin barrier function in the participants.

In recent years, products for sensitive skin have continuously emerged, and the common ingredients in these products are mainly minerals and plant extracts. An impaired skin barrier is one of the main mechanisms of sensitive skin, whose barrier function is mainly undertaken by the cuticle. Calcium ions are closely related to the division and differentiation of keratinocytes, as well as the barrier function of the epidermis. Yuspa et al. have reported that through keratinocyte cultures, high extracellular concentrations of calcium promote keratinocyte differentiation and stratification and glutamine transferase expression, formation of keratinocyte envelopes, and cellular differentiation indicators such as keratin 1, keratin 10 and filromerin; therefore, calcium is essential for the formation of the cuticle barrier [27]. In vitro experiments also revealed that if the isolated skin does not have sufficient calcium ions in the culture medium after the disruption of the barrier function, the calcium ion concentration and concentration gradient will not return to normal and subsequently delay the recovery of the barrier function [28, 29]. In this study, the participants with sensitive skin had significantly reduced TEWL after using a spray containing natural birch juice (Fig. 1), which may be due to the calcium ions in the birch juice that facilitate the repair of the skin barrier function.

Here, using the spray containing natural birch juice 4 weeks resulted in a gradual decrease in blood flow perfusion values (Fig. 3), and each value was significantly different from its baseline value (*P* < 0.05). Additionally, according to the images under cross-polarized light mode, both sprays have the effect of improving the degree of redness when used alone. The difference between the two groups was not statistically significant. Only positive control was set up and no negative control was set up, which is the limitation of this study. That is, negative controls were not used to eliminate the influence of variables such as environmental, seasonal and participants’ own changes on the results.

Yamasaki and Gallo have proposed that the innate immune system triggers inflammatory reactions and mediates symptoms of sensitive skin, resulting in skin redness and erythema [6, 30]. Spa therapy is an effective treatment for skin inflammation, and trace elements such as strontium and selenium may be the main effective elements of this therapy. Using a recombinant skin model, Kelerier et al. investigated the regulatory effects of strontium and selenium on inflammatory skin cytokines (IL-1α, TNF-α, and IL-6) and have reported that both strontium and selenium can effectively reduce the production of inflammatory cytokines in inflamed skin [31]. Birch juice contains strontium and selenium, which may be one of the reasons for its ability to reduce skin inflammation and redness.

The current perception threshold (CPT) of skin can be quantitatively measured by a CPT/C neurometer, which reflects the sensitivity of skin to stimulation. The lower the measured CPT value is, the more sensitive the sensory nerve of the skin is to the stimulus, and conversely, the less sensitive it is [25]. This study results revealed that after 2 and 4 weeks of spraying with the natural birch sap spray, the CPT value did not increase at 2000 Hz and 5 Hz. However, at 250 Hz, after 2 weeks and 4 weeks of spray application, the CPT value increased (Fig. 6), which indicated that the spray A reduced the sensitivity of skin sensory nerves to a certain extent. Studies have suggested that when strontium salt is used to treat faces with sensitive skin, the CPT value is significantly increased, indicating that strontium salt can improve skin sensitivity to stimulation [25]. In 1999, Hahn also reported that strontium salt can significantly reduce the sensory stimulation caused by some substances [32].

Sting sensation is considered to be one of the important characteristics of sensitive skin, so the sting sensation test is widely used in identifying sensitive skin [33]. The lactic acid sting test has been used as a method to identify facial skin sensitivity in many studies. The test is generally targeted at the nasolabial sulcus because this area has high permeability of the cuticle, high density of accessory organs, and a rich sensory neural network. The higher the LAST score is, the more sensitive the skin is; the lower the score is, the less sensitive the skin is [34, 35]. This study’s results indicated that after 2 and 4 weeks of using the spray A, the participants’ LAST scores decreased gradually (Table 3), and a significant difference was observed (*P* < 0.05) compared with baseline. The LAST score of people with sensitive skin decreased after using repair products have been reported in literature before [36, 37]. We found that the use of both sprays significantly reduced LAST scores, possibly because both sprays contain calcium and magnesium ions, resulting in enhanced epidermal barrier function in subjects with sensitive skin.

Moreover, the birch spray can reduce the sensory nerve sensitivity of the skin. A previous work by Eunyoung Lee et al. may explain the underlying mechanism behind these benefits [31].

It has been reported in literature that LAST scores positively correlated with TEWL, a* and EI value [38], were negatively correlated with stratum corneum hydration and current perception threshold (CPT) at 250 Hz [39]. The results of spray A in this study are consistent with the correlation demonstrated in the above literature. That is, the LAST score, TEWL and redness score decreased, CPT at 250HZ and hydration value increased during the 4 weeks of experimental period. Impaired skin barrier function is the main reason for sensitive skin [6]. When the barrier function of SC is impaired, it is less effective at preventing water from overevaporating, resulting in TEWL increases, stratum corneum hydration decrease [40], susceptibility to irritation enhanced (the scores of lactic acid sting incread). However, other factors may also have an impact, such as changes in the nervous system and/or epidermal structure. People with sensitive skin often have less hydration, more erythema and more skin with dilated distal blood vessels. From the preceding discussion, calcium ions in birch juice aids in the skin’s barrier functionality, and reducing TEWL and LAST score, increases and maintains the moisture content of the skin. Strontium salt of birch juice could improve skin sensitivity to stimulation and increase CPT value. And strontium and selenium could also reduce skin inflammation and redness.

Spray B was selected as the control in this study because thermal spring water has been widely reported to improve sensitive skin in several studies [41–44]. Spray A has a higher satisfaction rate than spray B, possibly because of its superior benefit in improving some symptoms of sensitive skin. As mentioned above, some spray A components, such as strontium and selenium, are minerals that reduce the production of inflammatory cytokines in inflammatory skin, reducing inflammation and redness. Calcium ions promote the repair of the skin barrier function. Therefore, Spray A may be more effective than Spray B in improving these symptoms and therefore has a higher satisfaction rate. However, we initially asked about overall satisfaction, which may make these results more positive. If we asked this question at the end of the subject’s self-assessment, they would have had the opportunity to review the shortcomings of the product in detail, and the overall satisfaction would have likely reduced. And this trial is a preliminary study, more research is needed to confirm this hypothesis.

There are two limitations to this experiment. First, we did not consider the impact of the environment on sensitive skin, that is, there was no negative control group. Our test site was Chengdu, Sichuan, China (102° 54′–104° 53′ E and 30° 05′–31° 26′ N), and the time was from mid-November to mid-December (average temperature was 6–12 ℃). This period just includes the transition from the end of autumn to the beginning of winter. Seasonal alternation, temperature change, sunlight, and other factors could aggravate sensitive skin [4, 45]. Cold environmental conditions could exert a negative effect on the skin. People exposed to severe weather in winter may experience dry and itchy skin, or their existing skin diseases may worsen [46, 47]. Therefore, the improvement effect of A and B may be masked by seasonal changes. The second was randomization without hierarchical grouping. The basic value distribution of TWEL in the two groups was unbalanced, and the difference was statistically significant. Thus, we adopted a mixed linear model for statistical analysis, and the differences in baselines would not affect the statistical results.

In future tests, we will comprehensively consider the influence of age, redness degree, TWEL, and other factors conducting stratified grouping. In addition, considering that sensitive skin is prone to relapse, it is necessary to extend the test time, include a suitably sized negative control group, and increase the number of cases.

## Conclusion

The birch spray is safe and effective for repairing sensitive skin, with efficacy and safety comparable to that of a widely accepted sensitive skin repair product. The results of the present study may provide a new option for the repair of sensitive skin.

## Data Availability

The data that support the findings of this study are available from the corresponding author upon reasonable request.

## References

[1] Draelos ZD (1997). Sensitive skin: perceptions, evaluation, and treatment. Am J Contact Derm.

[2] Sulzberger M, Worthmann AC, Holtzmann U (2016). Effective treatment for sensitive skin: 4-t-butylcyclohexanol and licochalcone A. J Eur Acad Dermatol Venereol.

[3] Misery L (2017). Neuropsychiatric factors in sensitive skin. Clin Dermatol.

[4] Inamadar AC, Palit A (2013). Sensitive skin: an overview. Indian J Dermatol Venereol Leprol.

[5] Misery L, Morisset S, Séité S (2021). Relationship between sensitive skin and sleep disorders, fatigue, dust, sweating, food, tobacco consumption or female hormonal changes: results from a worldwide survey of 10 743 individuals. J Eur Acad Dermatol Venereol.

[6] Berardesca E, Farage M, Maibach H (2013). Sensitive skin: an overview. Int J Cosmet Sci.

[7] Misery L, Weisshaar E, Brenaut E (2020). Pathophysiology and management of sensitive skin: position paper from the special interest group on sensitive skin of the International Forum for the Study of Itch (IFSI). J Eur Acad Dermatol Venereol.

[8] Misery L, Ständer S, Szepietowski JC (2017). Definition of sensitive skin: an expert position paper from the special interest group on sensitive skin of the international forum for the study of itch. Acta Derm Venereol.

[9] Achmon Y, Fishman A (2015). The antioxidant hydroxytyrosol: biotechnological production challenges and opportunities. Appl Microbiol Biotechnol.

[10] Smiljanic S, Messaraa C, Lafon-Kolb V (2022). Betula alba bark extract and empetrum nigrum fruit juice, a natural alternative to niacinamide for skin barrier benefits. Int J Mol Sci.

[11] Isidorov V, Szoka Ł, Nazaruk J (2018). Cytotoxicity of white birch bud extracts: perspectives for therapy of tumours. PLoS ONE.

[12] Softa M, Percoco G, Lati E (2019). Birch sap (betula alba) and chaga mushroom (inonotus obliquus) extracts show anti-oxidant, anti-inflammatory and dna protection/repair activity in vitro. J Cosmet Dermatol Sci Appl.

[13] Wnorowski A, Bilek M, Stawarczyk K (2017). Metabolic activity of tree saps of diferent origin towards cultured human cells in the light of grade correspondence analysis and multiple regression modeling. Acta Soc Bot Pol.

[14] Svanberg I, Sõukand R, Luczaj L (2012). Uses of tree saps in northern and eastern parts of Europe. Acta Soc Bot Pol.

[15] Ozolinčius R, Bareika V, Met R (2009). Chemical composition of silver birch (*Betula*
*pendula* Roth) and downy birch (*Betula*
*pubescens* Ehrh) Sap. Balt For.

[16] Ahtonen S, Kallio H (1989). Identification and seasonal variations of amino acids in birch sap used for syrup production. Food Chem.

[17] Kallio H, Ahtonen S, Raulo J (1985). Identification of the sugars and acids in birch sap. J Food Sci.

[18] Solano F (2020). Metabolism and functions of amino acids in the skin. Adv Exp Med Biol.

[19] Yang M, Zhou M, Song L (2020). A review of fatty acids influencing skin condition. J Cosmet Dermatol.

[20] Michalak M, Pierzak M, Bet K (2021). Bioactive compounds for skin health: a review. Nutrients.

[21] Reins RY, Hanlon SD, Magadi S (2016). Effects of topically applied vitamin d during corneal wound healing. PLoS ONE.

[22] Pullar JM, Carr AC, Vissers MCM (2017). The roles of vitamin C in skin health. Nutrients.

[23] Liu ML, Chun-Hui LV (2015). Application of vitamins and their derivatives in cosmetics. Jiangxi Chem Ind.

[24] Huang R (2012). Application of minerals in cosmetics. Deterg Cosmet.

[25] Lee E, An S, Lee TR (2009). Development of a novel method for quantitative evaluation of sensory skin irritation inhibitors. Skin Res Tech.

[26] Angelova-Fischer I (2016). Irritants and skin barrier function. Curr Probl Dermatol.

[27] Yuspa SH, Kilkenny AE, Steinert PM (1989). Expression of murine epidermal differentiation markers is tightly regulated by restricted extracellular calcium concentrations in vitro. J Cell Biol.

[28] Elias P, Ahn S, Brown B (2003). Origin of the epidermal calcium gradient: regulation by barrier status and role of active vs passive mechanisms. J Invest Dermatol.

[29] Elias PM, Wu Y, Chen C (2010). Regulation of epidermal barrier functions by calcium. J Clin Dermatol.

[30] Yamasaki K, Gallo RL (2009). The molecular pathology of rosacea. J Dermatol Sci.

[31] Celerier P, Richard A, Litoux P (1995). Modulatory effects of selenium and strontium salts on keratinocyte-derived inflammatory cytokines. Arch Dermatol Res.

[32] Hahn GS (1999). Strontium is a potent and selective inhibitor of sensory irritation. Dermatol Surg.

[33] Farage MA, Katsarou A, Maibach HI (2006). Sensory, clinical and physiological factors in sensitive skin: a review. Contact Derm.

[34] Seidenari S, Francomano M, Mantovani L (1998). Baseline biophysical parameters in subjects with sensitive skin. Contact Derm.

[35] Querleux B, Dauchot K, Jourdain R (2008). Neural basis of sensitive skin: a fMRI study. Skin Res Tech.

[36] Jeong S, Lee SH, Park BD (2016). Comparison of the efficacy of atopalm multi-lamellar emulsion cream and physiogel intensive cream in improving epidermal permeability barrier in sensitive skin. Dermatol Ther (Heidelb).

[37] Zhang Y, Jin Y, Humbert P (2021). An herbal cream reduces erythema of sensitive skin. J Cosmet Dermatol.

[38] Pan Y, Ma X, Song Y (2021). Questionnaire and lactic acid sting test play different role on the assessment of sensitive skin: a cross-sectional study. Clin Cosmet Inv Derm.

[39] Jiang W, Wang J, Zhang H (2022). Seasonal changes in the physiological features of healthy and sensitive skin. J Cosmet Dermatol.

[40] Berardesca E, Maibach HI (1990). Transepidermal water loss and skin surface hydration in the non invasive assessment of stratum corneum function. Derm Beruf Umwelt.

[41] Ferreira MO, Costa PC, Bahia MF (2010). Effect of São Pedro do Sul thermal water on skin irritation. Int J Cosmet Sci.

[42] Zeichner J, Seite S (2018). From probiotic to prebiotic using thermal spring water. J Drugs Dermatol.

[43] Merial-Kieny C, Castex-Rizzi N, Selas B, Mery S, Guerrero D (2011). Avène Thermal Spring Water: an active component with specific properties. J Eur Acad Dermatol Venereol.

[44] Bieber T (2011). More scientific evidence for the therapeutic benefit of hydrotherapy in Avène. J Eur Acad Dermatol Venereol.

[45] Berardesca E, Farage M, Maibach H (2013). Sensitive skin: an overview. Indian J Dermatol Venereol Leprol.

[46] Engebretsen KA, Johansen JD, Kezic S, Linneberg A, Thyssen JP (2016). The effect of environmental humidity and temperature on skin barrier function and dermatitis. J Eur Acad Dermatol Venereol.

[47] Brenaut E, Barnetche T, Gall-Ianotto CL (2020). Triggering factors in sensitive skin from the worldwide patients' point of view: a systematic literature review and meta-analysis. J Eur Acad Dermatol Venereol.

